# “*We needed this*”: perspectives of parents and healthcare professionals involved in a pilot newborn screening program for spinal muscular atrophy

**DOI:** 10.1016/j.eclinm.2021.100742

**Published:** 2021-02-19

**Authors:** Didu S.T. Kariyawasam, Arlene M. D'Silva, Janine Vetsch, Claire E. Wakefield, Veronica Wiley, Michelle A. Farrar

**Affiliations:** 1 Department of Neurology, Sydney Children's Hospital, Randwick, Sydney, New South Wales, Australia; 2 School of Women's and Children's Health, University of New South Wales Medicine, UNSW Sydney, New South Wales, Australia; 3 Kids Cancer Centre, Sydney Children's Hospital, Randwick, Australia; 4 NSW Newborn Screening Program, Children's Hospital Westmead, Westmead, New South Wales, Australia; 5 Faculty of Medicine and Health, University of Sydney, Sydney, New South Wales, Australia

## Abstract

**Background:**

Newborn screening (NBS) for spinal muscular atrophy (SMA) is a recognised model through which health outcomes can be improved. However, perspectives of parents and healthcare professionals (HCPs) involved in such programs are largely unknown.

**Methods:**

A pilot program for SMA ran from August 2018-July 2020. Using a mixed-methods convergent methodology, we used a self-administered questionnaire to understand parents’ perceptions and psychological impact of the program from diagnosis to treatment. We thematically analysed successes/challenges encountered by HCPs and recommendations for service improvement from both participant groups.

**Findings:**

202,388 infants were screened for SMA and the perceptions of 44 parents and HCPs affected by a positive result in eighteen newborns was ascertained. Parents (n=29, 100%) were satisfied with NBS for SMA. Although screen-positive result was distressing for all parents, quality of life improved over time [CarerQoL-7D baseline median score 4 (SD=1.4) vs six-month median score 8 (SD=1.3), p<0.001)]. Challenges for HCPs included managing the time-critical nature of the pathway whilst remaining cognisant of limitations associated with the predictive screening test.

**Interpretation:**

Interpretation: NBS for SMA fulfils criteria for population-wide screening. Net benefits are acknowledged by stakeholders to optimise lifelong outcomes. Harms including psychological distress associated with a screen-positive result may be managed by targeted psychosocial support, information provision and a personalised model of care together strengthening healthcare systems.

**Funding:**

The NSW Pilot NBS study was funded by Luminesce Alliance. Dr Kariyawasam received funding from the RTP Scholarship, University of New South Wales and The Freedman Family Foundation Scholarship, Sydney Children's Hospital Foundation.

## 1 Introduction

Spinal Muscular Atrophy (SMA), characterised by progressive loss of motor neurones of the brainstem and spinal cord, culminates in muscle weakness and wasting [1]. In its severest and most frequent form, progressive infantile paralysis and premature death before two years of age occurs [1]. Known as an irreversible neurodegenerative disorder, public acceptability of newborn screening (NBS) for the condition was previously limited due to the lack of a disease-modifying intervention [2].

The advent of the first approved and licenced therapeutic agents for SMA has shifted the paradigm of management for affected children from supportive to proactive care [3]. Early (particularly presymptomatic) therapeutic intervention has shown to improve survival, reduce morbidity and facilitate functional motor skill development [4]. Well recognised delays in making a clinical diagnosis [5] coupled with the necessity for early intervention have prompted international consensus opinions that NBS for SMA should be established to enable early diagnosis, clinical decision-making and treatment, thus minimising the extent of irreversible motor neurone loss [6]. Australia initiated a two-year state subsidised NBS pilot program for SMA in New South Wales (NSW) and the Australian Capital Territory (ACT) on August 1^st^, 2018. Results from the first data cut in August 2019 highlighted the accuracy, efficiency and short-term health outcomes of the screening and diagnostic pathway [7].

Studies thus far have concentrated solely on laboratory capabilities and clinical implementation of NBS processes for SMA [7]. Whilst the feasibility and accuracy of these genomic methodologies are undoubtedly important, the 2018 Newborn Bloodspot Screening National Policy Framework advocates a family-focussed approach to supporting parents and prioritises understanding families’ perspectives of such programs [8]. Experience of information provision, access to appropriate resources and quality of health-provider communication are important areas for consideration when developing and refining professional guidelines regarding screening for rare conditions, requiring formal assessment [9]. This is especially relevant as SMA is one of the first conditions to be detected using novel high through-put genomics within a (newborn) screening strategy [10].

Previous research with the general public details the hypothetical benefits of implementing a NBS program for SMA [2], as well as revealing uncertainties associated with achieving early diagnosis [11]. This is particularly relevant for newborns who may not develop infantile onset disease, and therefore are at risk of over-surveillance or over-treatment secondary to a neonatal screening result [11]. Concerns about the potential for psychosocial disruption to familial bonds from NBS programs have focused on parents receiving false-positive results [12]. There are few studies to date that have prospectively explored the psychosocial sequalae on families with genetic true-positive screen results after NBS, with no literature on the early impact of NBS for SMA. An understanding of the perceived benefits and disadvantages of such programs from parents’ perspectives will ascertain whether hypothetical concerns align with real-world experiences.

Healthcare professionals (HCPs) play an integral role in information provision and facilitation of parental decision making throughout the pathway. HCPs acknowledge barriers to maximising parental understanding when disclosing screening/diagnostic results for common genetic conditions whilst supporting parents to manage their distress [13]. The perspectives of HCPs involved in NBS programs for rare and complex genetic conditions such as SMA have not yet been investigated.

As the first study to assess perspectives and recommendations from a parent and healthcare professional viewpoint for NBS in SMA, we envisage that our findings will be useful to inform best clinical practice and optimise clinical translation of new genomic technologies and NBS services for SMA and other rare diseases world-wide.

This study aimed to evaluate:

Parents’ perceived benefits/disadvantages associated with taking part in the NBS program for SMA, including satisfaction and impact on quality of life (QoL). Parents’ perceptions on quality and clarity of information provision, communication and access to appropriate healthcare and psychosocial support from pre-screening, screening to diagnostic result disclosure. HCPs’ perceived successes and challenges of healthcare provision. Recommendations regarding how to address unmet areas of needs from both parents’ and HCPs’ perspectives.

## 2 Methods

### 2.1 Clinical pathway for the pilot NBS program for SMA

**Fig. 1:**
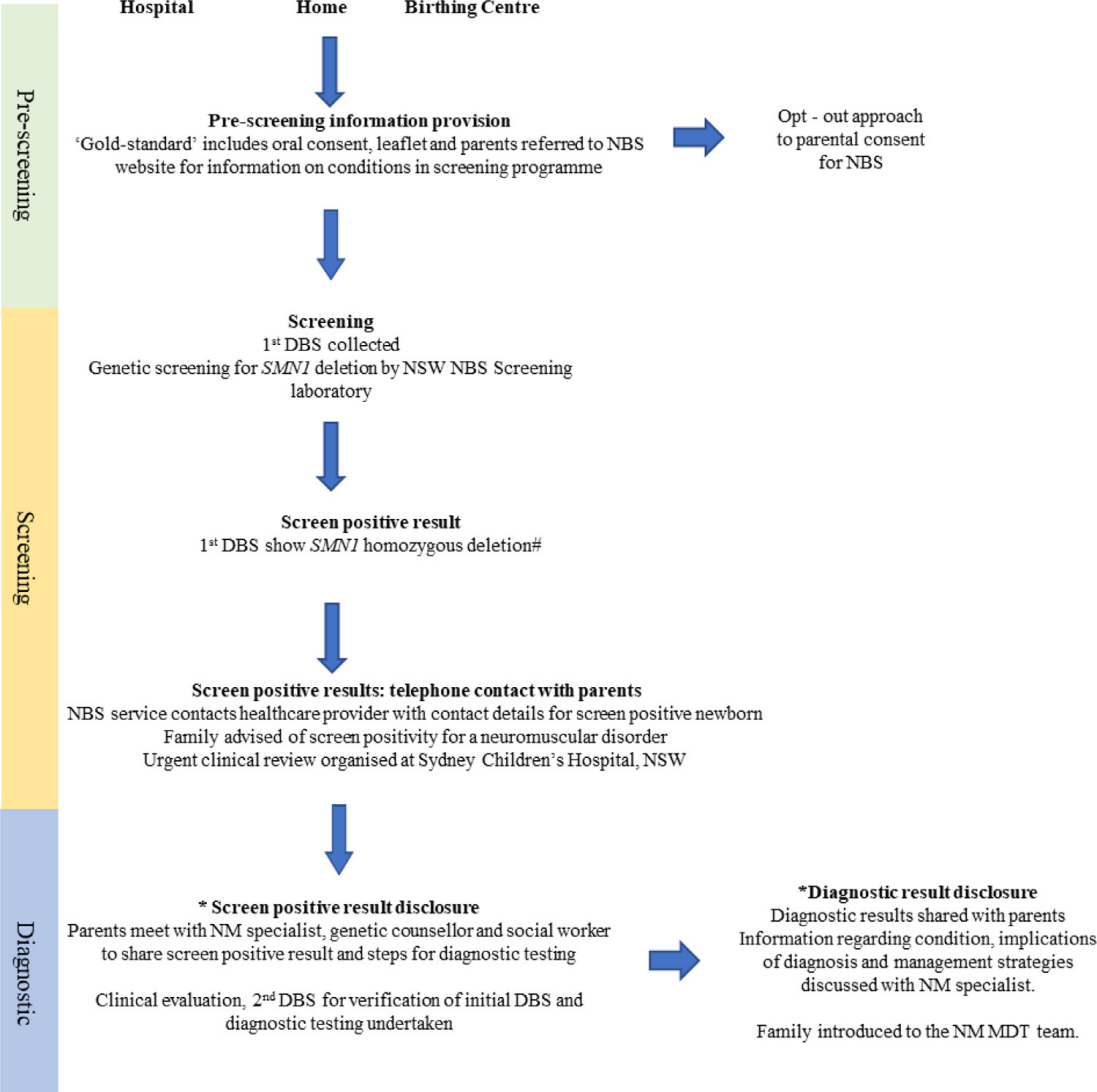
The clinical pathway from pre-screening, screening to diagnosis in the pilot newborn screening for spinal muscular atrophy. DBS (dried blood spot), MDT (multidisciplinary), NBS (newborn screening), NM (neuromuscular), NSW (New South Wales) *Parents invited to participate in the study. # The screening pathway evolved during the pilot study such that all infants with 0 *SMN1* were all screened positive. The rationale for this was to enable proficiency testing. Consequently, *SMN2* copy number did not determine screen positivity in this pilot and a single individual with 4 *SMN2* was identified in the second year.

The pilot program centred on a three-part process, including pre-screening, screening, and diagnostic stages for screen-positive newborns (Figure 1). The screening pathway evolved during the pilot study such that all infants with *0 SMN1* were all screened positive. The rationale for this was to enable proficiency testing. Consequently, *SMN2* copy number did not determine screen positivity in this pilot and a single individual with 4 *SMN2* was identified in the second year.

### 2.2 Participants

**Fig. 2:**
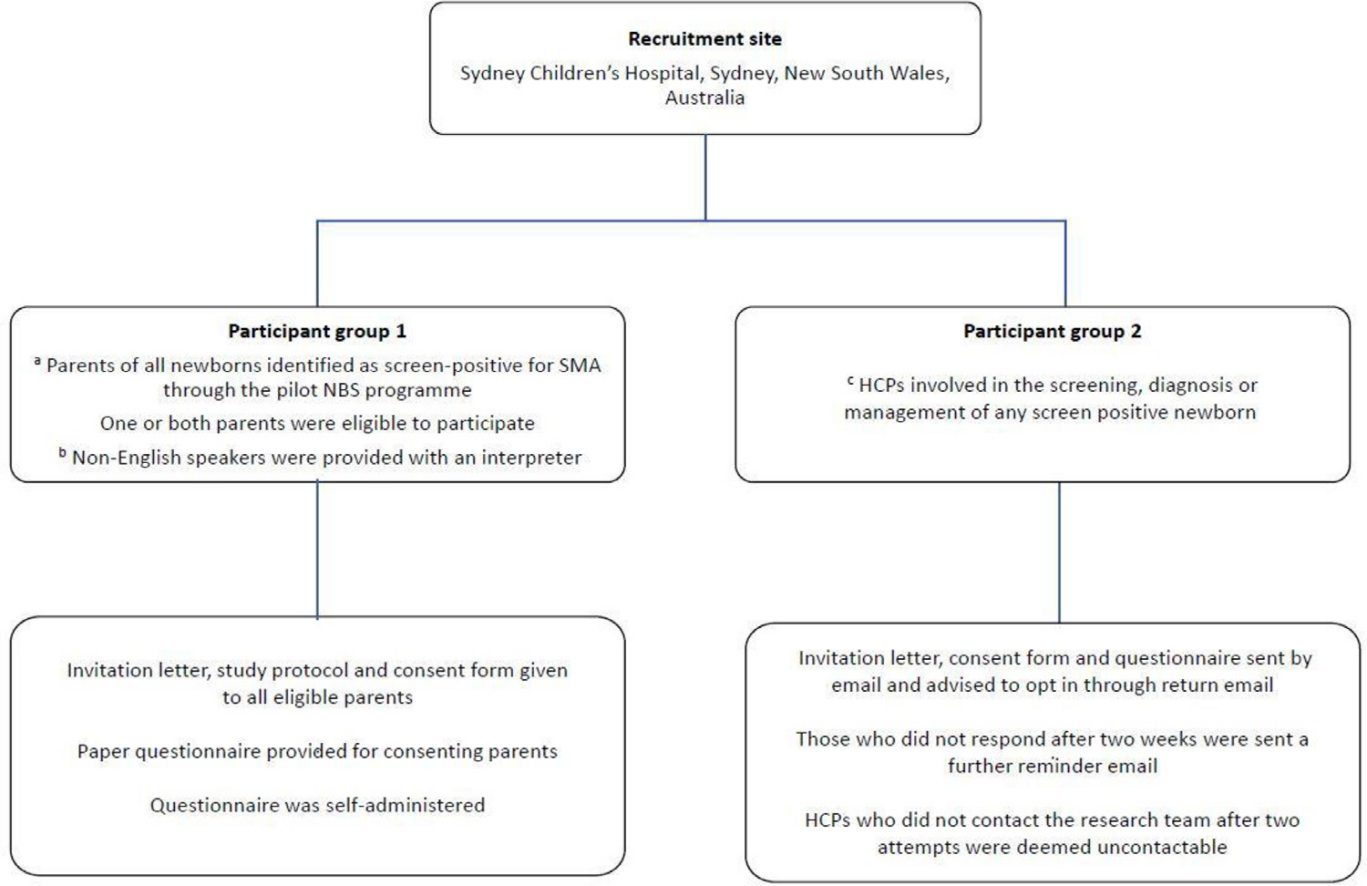
Study site, participant, and recruitment methodology for the study ^a^ Exclusion criteria included parents of newborns with clinical identification of SMA without NBS or those not screened (n = 1). ^b^ We interviewed Non-English speakers with the assistance of a face-to-face or phone interpreter, using the same questionnaire and transcribed answers and phrases verbatim. ^c^ We identified eligible healthcare professionals (HCPs) through the state-wide newborn screening registry (that collates information on screen-positive newborns and the corresponding designated HCP named on the dried blood spot) and through HCPs within the specialist neuromuscular network across Sydney Children's Hospital Network.

Participants were recruited into the study using a whole population sampling method that involved examining the entire population of newborns in NSW/ACT Australia between 1 August 2018 and 31 July 2020. This region approximates 1/3 of all births nationally and NBS uptake is extremely high with over 99% of live births screened. The inclusion criteria comprised parents of all newborns identified as screen positive for SMA. They were invited to participate in the study following diagnostic confirmation whilst management plans were being implemented . Non-English speakers were assisted with a face-to-face or phone interpreter (Figure 2). Eligible parents provided verbal and written informed consent to take part in the study. Exclusion criteria included parents of newborns with clinical identification of SMA without NBS or those not screened, parents with severe depression or psychosis as determined by the judgemental of the doctors or with significant difficulties that would impact on their ability to complete the questionnaire with assistance e.g. significant cognitive difficulties or parents with significant substance abuse.

The inclusion criteria for HCPs (clinicians and allied health professionals) comprised those involved in the screening of individuals through the NBS program or in the diagnosis and/or management of screen positive newborns within the specialist neuromuscular team across Sydney Children's Hospital Network. Clinicians within this cohort included neurologists, paediatricians, neonatologists, geneticists, scientists. Allied health professionals included physiotherapists, genetic counsellors, psychologists' social workers and nurses

Participant inclusion criteria and recruitment methodology are detailed in Figure 2. The study was approved by the Sydney Children's Hospitals Network Human Research Ethics Committee (LNR/18/SCHN/307).

### 2.3 Study measures

We used a mixed-methods approach to investigate parent experiences of NBS for SMA at each stage using a convergent parallel design where qualitative and quantitative data were collected concurrently [14]. This methodology has successfully been used to explore family perspectives to genetic population screening in SMA [15]. We used a self-administered questionnaire which included quantitative measures and open-ended questions. Qualitative items were evaluated using the 5-point Likert scales and were then dichotomized. Experts with knowledge of qualitative research, NBS and managing children with SMA developed the questionnaire (Table S1 and S2). QoL for parents was assessed using the CarerQoL-7D [16], completed at baseline and six months from screen-positive result disclosure, with higher scores indicating a better QoL. The HCP questionnaire included 16-items to explore perspectives on service provision within the NBS program for SMA (Table S3).

### 2.4 Data Analysis

We analysed the data using a convergent design [14]. We analysed quantitative items using descriptive statistics in the Statistical Package for the Social Sciences version 12 (SPSS) whilst quantification of themes arising from the qualitative data occurred concurrently. A paired t-test was used to assess CarerQol-7D over a six-month period. Two researchers (AD and DK) familiarised themselves with the qualitative responses provided in the open-ended questions and manually coded these line by line. Double coding occurred in 50% of questionnaires in each participant group and interrater reliability utilising percentage agreement methodology showed a high degree of agreement (97%) between the two researchers. A coding tree was generated by researchers AD and DK and refined by researcher DK. Researchers DK and AD identified new themes out of the codes using an inductive thematic approach. We discussed and refined themes iteratively until consensus was reached. Themes were used to complement quantitative results, using illustrative quotes to highlight findings.

### 2.5 Role of funding source

The NSW Pilot NBS study was funded by Luminesce Alliance. Dr Kariyawasam received funding from the RTP Scholarship, University of New South Wales and The Freedman Family Foundation Scholarship, Sydney Children's Hospital Foundation. Funders did not have a role in the design of the study or the interpretation of the results.

## 3 Results

202,388 infants were screened for SMA and the perceptions of 44 parents and HCPs directly affected by a positive result in eighteen newborns was ascertained. This included twenty-nine out of 36 eligible parents (response rate 81%). At least one parent of each of the screen-positive newborns participated and for parents with one response, the non-participant indicated they were in alignment with their partner and did not complete a separate survey. Fifteen out of 18 eligible HCPs consented to participate (response rate 83%). HCPs that did not respond to two email reminders were deemed ineligible to participate. Parents identified with a spectrum of ethnicities/religions and came from a variety of education/social backgrounds (Table 1). No parent had a previous child with SMA. There was a family history of SMA for 1/29 (3%) of the parent cohort. There was a spectrum of clinical presentations amongst the newborn cohort and a range of different therapeutic avenues were pursued. The therapeutic avenues included approved treatments i.e. nusinersen or treatment with novel therapeutics including Zolgensma and Risidiplam, accessed through clinical trials or compassionate use programs.

**Table 1:** Demographic and clinical characteristics of screen-positive newborns, parents and healthcare professionals.

Characteristics of newborns (N = 18)	
	n (%)
**Sex of newborn**	
Male	8 (44%)
Female	10 (56%)
*SMN2 copy number	
**2*SMN2***	10 (58.8%)
**3*SMN2***	6 (35%)
**4 *SMN2***	1 (5.9%)
**Clinical status at time of questionnaire**	
Presymptomatic	11 (61%)
Symptomatic	6 (33%)
Not confirmed to have SMA on diagnostic testing	1 (6%)
**Clinical management**	
Therapeutic intervention with disease modifying agent	14 (77%)
Monitoring for symptom onset prior to therapeutic intervention	2 (11%)
Palliation	1 (6%)
Not confirmed to have SMA on diagnostic testing	1 (6%)
**Characteristics of parents (N = 29)**	
**Age of parent (median, range and standard deviation) in years**	31 (17-50) SD 8.08
	**n (%)**
**Gender**	
Female	17 (59%)
Male	12 (41%)
**Country of origin**	
Australia	12 (41%)
Other	17 (59%)
**Primary language**	
English	15 (52%)
Other	14 (48%)
**Religion**	
Monotheistic (Christianity/Islam/Judaism)	20 (69%)
Polytheistic (Hinduism/Buddhism)	4 (14%)
No religion/other	5 (17%)
**Highest educational level**	
Secondary (Year 10 and below)	5 (17%)
Secondary (Year 11&12)	4 (14%)
Tertiary (including certificate/diploma, university/postgraduate degree	20 (69%)
**Prior knowledge of genetics and NBS programs**	
Parent has participated in classes on genetics	4 (14%)
Parent has participated in previous NBS programs	19 (66%)
**Characteristics of healthcare professionals (N = 15)**	
**Profession**	
a Clinician	9 (60%)
b Allied health professional	6 (40%)
**Length of time spent in designated profession**	
< 5 years	1
years	2
11-20 years	5
> 20 years	7

a Clinicians included neurologists (n = 5), paediatricians, neonatologists (n = 1), geneticists (n = 2), scientists (n = 1)

b Allied health professionals included physiotherapists (n = 1), genetic counsellors (n = 1), psychologists’ social workers (n = 1) and nurses (n = 3)

⁎ *SMN2* copy numbers in n = 17 participants (the false positive is removed from the denominator). The false positive screen was resolved by retesting using a different assay.

### 3.1 Parents’ perceived benefits/disadvantages, including satisfaction and impact on quality of life

All 29 parents (100%) reported that they would participate in future NBS programs for SMA. Parents (including those who opted not to treat or to monitor for symptom emergence prior to intervention) described an overall 100% satisfaction rate with the program. Importantly, 21/29 (72%) parents spontaneously advocated for equitable access to NBS for SMA across Australia. Perceived benefits of taking part in the program included themes of *cherishing their child and family, early access to management options, potential of better clinical outcomes* and *facilitating the diagnostic journey for the family* (Table 2).

**Table 2:** Parents perceived benefits and disadvantages and health care professionals perceived successes and challenges associated with newborn screening for spinal muscular atrophy, with illustrative quotes.

PARENTS’ PERSPECTIVES	HEALTHCARE PROFESSIONAL PERSEPCTIVES
Benefits	Successes
**Cherishing their child and family** *It's really hard carrying him for nine months then maybe having to say goodbye soon but knowing the diagnosis has made us concentrate on enjoying the little things, each smile, each laugh (mother of symptomatic newborn, 2 SMN2).*	**Early diagnosis and implementing intervention strategies** *We recognized evolution of symptoms in some neonates with 2 SMN2 copies while treatment was being initiated in the first four weeks. The times-scale to initiate treatment is best achieved through NBS and the challenge is to establish models of care to facilitate this (clinician).*
**Access to early management options and potential to see improvement in clinical outcomes** *Even though we didn't end up treating, we needed to have access to all the options so we could make the best choices. (father of symptomatic newborn, 2 SMN2).* *We needed this. Even if there was no treatment I would still want to know [*the diagnosis*] because there is hope for other things to be done to help also (mother of presymptomatic newborn, 2 SMN2).*	**Equitable diagnosis and accessing health resources, within a personalized model of care** *Simultaneous provision of quality care at personalized and population level. I recognized differences in sociodemographic characteristics, parental reactions and information/support needs especially for rural/remote families (clinician).* *Equal opportunity for families to gain diagnosis that is independent from the expertise of staff and location. Rural hospitals participating in the NBS allows families the safe access to medical results, comparable to metropolitan based families (allied health professional).*
**Disadvantages**	**Challenges**
**Fear for the future** *I'm just really anxious. I keep standing over [the baby] and watching because of the diagnosis. I guess I'm not treating [*him*] like a normal baby (mother of presymptomatic newborn, 2 SMN2).* *It's put a huge strain on us (as partners) as we're dealing with the result in different ways (mother of symptomatic newborn, 2 SMN2).*	**Managing the timing of information provision, assessment, and intervention** *Although the treatment is so time critical, giving parents the time to process the diagnosis is important (clinician).* *There is a need for very rapid education [*for parents*] in genetics and you have to develop trust with the family very quickly, in order to intervene quickly enough (clinician).*
**Potential for stigmatization** *This is a big result in our culture. People in our community may look at him and us differently and we have to contend with that. Especially as it is genetic (mother of presymptomatic newborn, 2 SMN2).*	**Understanding, translating and relating unexpected findings** *The equivocal genetic result was very difficult for the family and for us. I think the process for following up on such unexpected results needs to be streamlined (clinician).* *It can be difficult managing parents’ feelings when an unexpected result arises (allied health professional).*
	**Managing uncertainty associated with using predictive screening tests** *There is uncertainty for infants with >3 SMN2 copy numbers – families will respond differently to this, so a provision of personalized care is important (clinician).* *It's a challenge (for us and the families) to predict exactly how a baby will be with the screen result. Living with this uncertainty can be difficult. Sometimes families can't see the advantage in treating as their baby has no visible symptoms of disease (allied health professional).*

Perceived disadvantages associated with receiving a screen-positive result included a theme of *fear for the future*. One individual commented on the potential for stigmatisation from the wider community (Table 2).

No parent expected a screen-positive result for their newborn or anticipated anxiety arising from the program at pre-screening. On receiving a screen-positive result, the majority of parents reported that they experienced anxiety (n=24;83%), although concomitant feelings of anger (n=4, 14%), sadness (n=4, 14%) and/or confusion (n=10, 34%) were also experienced. In 48% of parents (n=14), a period of ‘hopefulness for a ‘false-positive result’ ensued between screening and diagnostic stages, particularly as the newborn appeared seemingly healthy to parents: “*I remember thinking he hasn't got this, my baby is so well…” (mother).* Whilst anxiety and sadness remained as parents transitioned to diagnostic and care planning stages, in 28% (n=8) this was balanced with emergence of positive emotions including ‘hopefulness’, ‘confidence’ and ‘reassurance’. Quality of life for parents improved significantly over time [baseline median score=4 (SD=1.4, IQR = 1.25) vs six-month median score=8 (SD=1.3, IQR = 1.75), p < 0.001)].

### 3.2 Parents’ perceptions on quality and clarity of information provision, communication, and access to healthcare resources

**Fig. 3:**
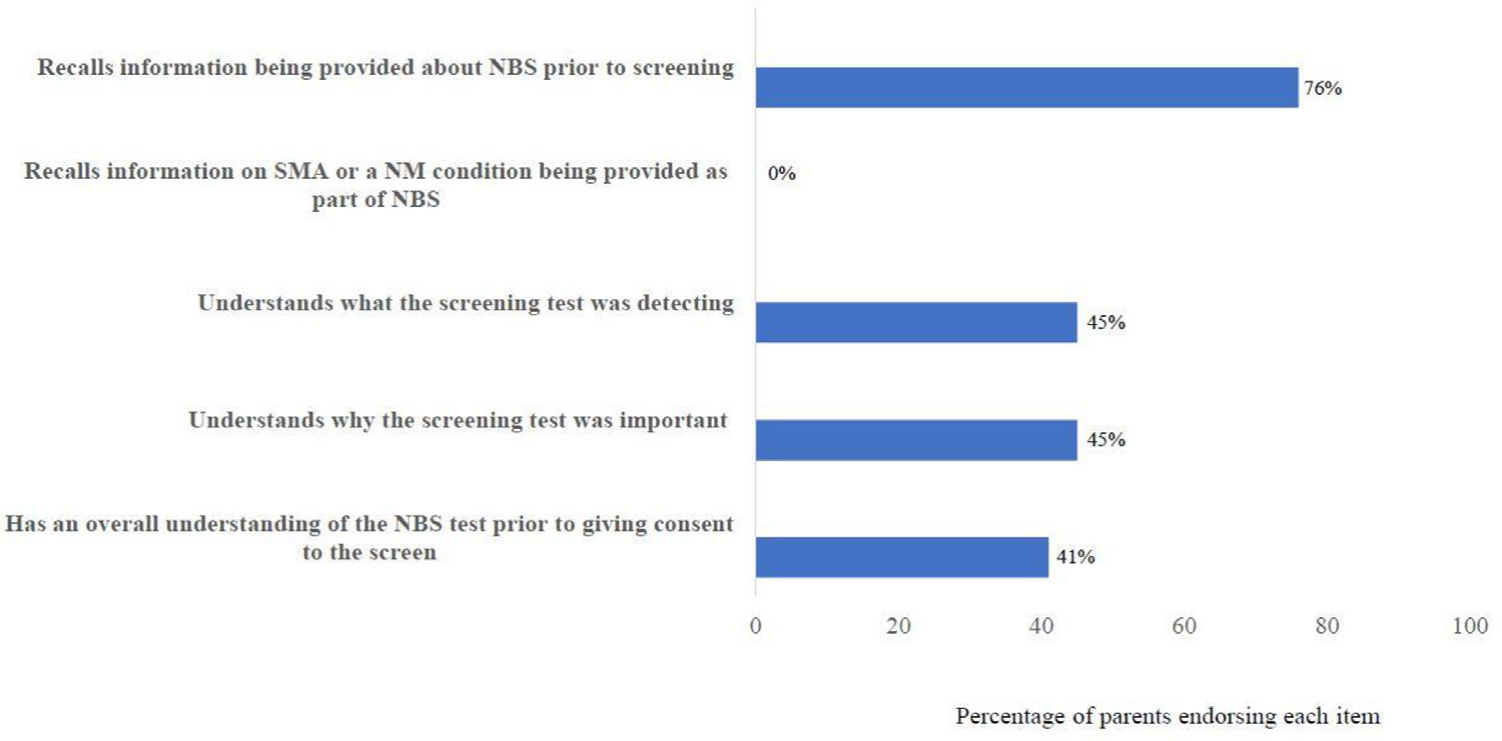
Parents’ perceptions on their recall and understanding of information provided during the pre-screening stage of newborn screening for spinal muscular atrophy. Data was collected from (n = 29) parents using the 5-point Likert scales and were then dichotomized into “Extremely and Very true” (represented in the Figure) and “Somewhat, A little and Not at all true”. The x-axis represents percentage of parents from the total cohort endorsing each item.

Among the 29 parents, 22 (76%) recalled receiving general information by maternity healthcare workers about NBS before screening (Figure 3). No parent recalled information relating to screening for a neuromuscular condition and over half the cohort (n=16; 55%) felt they did not understand the content or process of NBS screening prior to opting for the procedure. Nine (31%) parents described a false sense of reassurance because they had undergone prenatal/preconception testing, with normal results; “*Our prenatal tests were all fine, so the [*NBS result*] was a complete surprise. I don't understand why this didn't come up” (father).*

Thirteen parents (45%) perceived that receiving disease-specific information at pre-screening could have reduced the psychological distress associated with a screen-positive result. One father described, “*I didn't even know [*SMA*] was being tested so [*I*] was totally confused and underprepared when I got the results. The lack of information made the shock so much worse.*”

**Fig. 4:**
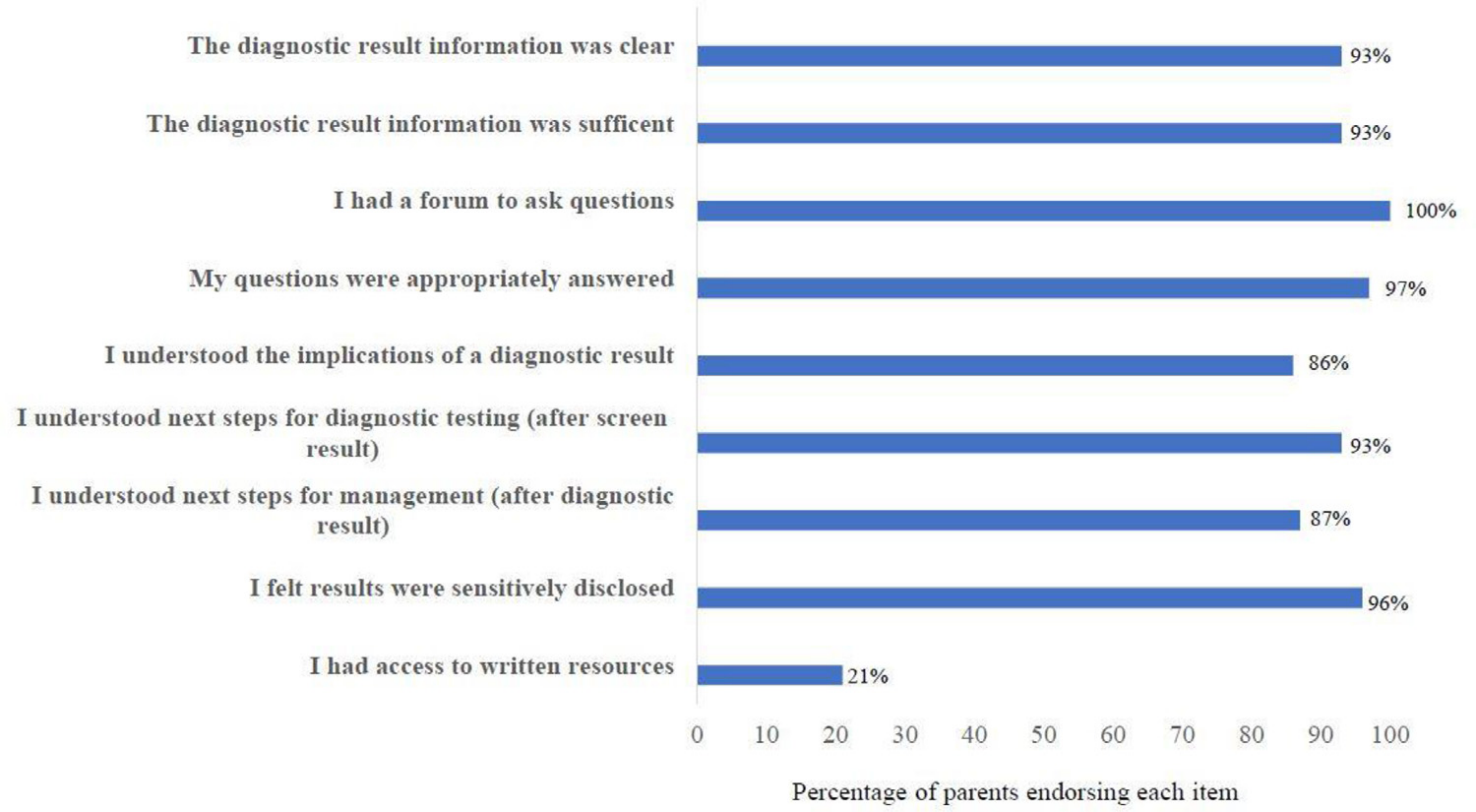
Parents’ perceptions on the process of diagnostic result disclosure during the newborn screening pathway for spinal muscular atrophy. Data was collected from (n = 29) parents using the 5-point Likert scales and were dichotomized into “Extremely and Very true (represented in the Figure) and “Somewhat, A little and Not at all true’. The x-axis represents percentage of parents from the total cohort endorsing each item

Of 29 parents, 19 (66%) felt that the information provided when first receiving notice of a screen-positive result was clear and 17 (59%) felt that it was sufficient. Median time from receiving a screen positive result for SMA to specialist review was 1 day. Most parents reported feeling more informed after discussing results with the specialist team, including understanding next steps for diagnostic testing (after receiving screen results) and management options after diagnostic confirmation of SMA (Figure 4). Over half (n=17; 58%) of the cohort spontaneously advocated for expedient access to specialist expertise. One mother emphasised “*With this rare diagnosis, you need to be surrounded by people who have knowledge in this specific area, otherwise you are not getting the most up-to-date advice and management*”.

However, 41% (n=12) described barriers to comprehending the information including their own heightened emotional response and/or the complexity of this rare and previously unheard-of condition, stating “*I couldn't understand the condition as it was completely new. It's a complicated disease to understand, especially the genetics of it. I couldn't take all the information in as I was so shocked”* (*mother*).

Perhaps as a further consequence of these barriers to information provision, over half of parents (17/29;58%) detailed a period of ‘information seeking’ between receiving screen and diagnostic results, with variable results, “*I was told not to, but I looked on the internet…and it was awful. They showed you the worst-case scenarios of [*untreated*] children*” (*mother*).

Of the cohort, 15 (52%) parents felt that the quality of HCP communication influenced their experiences of the healthcare system. One mother stated, “*The neuromuscular expert was brilliant. She took her time and really explained the diagnosis and management. Even though I was still devastated, we could look forward to planning the best care.*” Shared decision-making around next steps was important for some families, even when disease-modifying treatments were not instigated, “*I want my baby to have no pain. The* [neuromuscular team] *helped us come to the right path for our family. We were given all the options and in not treating we felt supported”* (*father*).

### 3.3 Healthcare professionals’ perceived successes and challenges of healthcare provision

Perceived successes of the program were synthesised into two major themes. Out of 15 HCPs, 87% (n=13) described a theme of *enabling early diagnosis and implementation of intervention strategies,* describing that NBS for SMA was a crucial platform from which to achieve timely diagnosis and initiate a therapeutic pathway. Particularly six HCPs (40%), all of whom had experience managing children with SMA in the pre-treatment era, acknowledged significant potential for improved clinical outcomes through NBS (Table 3). Four HCPs (27%) described a theme of e*quitable diagnosis and accessing health resources, within a personalized model of care.* Here*,* HCPs acknowledged that NBS circumvented potential barriers to achieving a timely diagnosis secondary to variability in health literacy, family psychosocial status and geographical location. In tandem with facilitating equity of access, six HCPs (40%) valued how NBS for SMA could promote access to individualised therapies for the newborn and targeted psychosocial support for families.

**Table 3:** Health care professionals perceived successes and challenges associated with newborn screening for spinal muscular atrophy, with illustrative quotes.

Successes	
Theme	Illustrative quotes
Early diagnosis and implementing intervention strategies	*Children can be treated as early as possible to produce the best possible outcomes… [NBS] allows us to intervene at the point that makes the most difference to the child (clinician).* *Early diagnosis, education and initiation of treatment has a dramatic effect on outcome. This can be achieved through NBS (clinician).* *We recognized evolution of symptoms in some neonates with 2 SMN2 copies while treatment was being initiated in the first four weeks. The times-scale to initiate treatment is best achieved through NBS and the challenge is to establish models of care to facilitate this (clinician).* *Through NBS, we provide treatment early and parents do not have to walk the journey of misdiagnosis or false reassurance when they have concerns for their baby (allied health professional).*
Equitable diagnosis and accessing health resources, within a personalized model of care	*Simultaneous provision of quality care at personalized and population level. I recognized differences in sociodemographic characteristics, parental reactions and information/support needs especially for rural/remote families (clinician).* *Equal opportunity for families to gain diagnosis that is independent from the expertise of staff and location. Rural hospitals participating in the NBS allows families the safe access to medical results, comparable to metropolitan based families (allied health professional).*
**Challenges**	
Managing the timing of information provision, assessment, and intervention	*Although the treatment is so time critical, giving parents the time to process the diagnosis is important (clinician).* *There is a need for very rapid education [*for parents*] in genetics and you have to develop trust with the family very quickly, in order to intervene quickly enough (clinician).*
Understanding, translating and relating unexpected findings	*The equivocal genetic result was very difficult for the family and for us. I think the process for following up on such unexpected results needs to be streamlined (clinician).* *It can be difficult managing parents’ feelings when an unexpected result arises (allied health professional).*
Managing uncertainty associated with using predictive screening tests	*There is uncertainty for infants with >3 SMN2 copy numbers – families will respond differently to this, so a provision of personalized care is important (clinician).* *It's a challenge (for us and the families) to predict exactly how a baby will be with the screen result. Living with this uncertainty can be difficult. Sometimes families can't see the advantage in treating as their baby has no visible symptoms of disease (allied health professional).* *It's a challenge to inform families of a diagnosis when time of onset [*of disease*] may be unclear especially as many may not develop symptoms for some time (clinician).*

Despite these positive experiences, HCPs described challenges associated with NBS for SMA. Five HCPs (33%) acknowledged challenges *managing the timing of information provision, assessment, and intervention*. The time critical nature of motor neuron loss in SMA contributed to perceived feelings of pressure to simultaneously confirm a diagnosis, share results with the family, assess the newborn and start on a therapeutic pathway. Just over half of HCPs (8/15;53%) described challenges in *understanding, translating, and relating unexpected findings.* The complexity of SMA genetics meant that understanding equivocal results, re-testing on different genomic platforms and interpreting these findings within a clinical context required a multidisciplinary approach. Six HCPs (40%) recognised difficulties in *managing uncertainty associated with using predictive screening tests*. For these HCPs, the two-tier screening test that uses *SMN2* copy number to facilitate a prognosis for the newborn had limitations, including uncertainty in the timing of disease-onset and severity of phenotype and difficulties relaying this uncertainty to parents.

### 3.4 Recommendations on how to address unmet areas of need

Parents and HCPs described a range of recommendations to address unmet needs. Themes centred around *empowering parents, expediting access to specialist care, and optimising reproductive choice* (Table 4). Some parents (6/29; 21%) felt that reproductive choice should be optimised by antenatal screening methods including access to preconception and prenatal testing for this condition.

**Table 4:** Parents’ recommendations on improvements to the pilot newborn screening program for spinal muscular atrophy, with illustrative quotes.

Theme	Subtheme	Illustrative quotes
**EMPOWERING PARENTS**	Parent and family-centred information provision	*I think just being told something, its difficult to retain. Some parents may not want to read information and its up to them, but I would have liked a reference material (father of presymptomatic newborn, 3 SMN2).* *Just after you've had a baby, you can't remember anything. I think something this important should be discussed in the antenatal period either in classes or by the GP (mother of presymptomatic newborn, 3 SMN2).* *I needed an understanding in my own language (mother of presymptomatic newborn, 2 SMN2).*
	Optimising content of information provided	*I needed information about testing for SMA specifically (mother of presymptomatic newborn, 3 SMN2).* *Its hard to get the correct amount of information but parents need to know what they are consenting to so you need to build that in (mother of presymptomatic newborn, 2 SMN2).*
	Accessing written resources	*I think if you had a written sheet, maybe it would stop families from going onto the internet and searching for answers from out of date sites (father of presymptomatic newborn, 2 SMN2).* *There is a lot of information to take in all at once - the genetics, the treatment etc. We need something that we can go back and refer to, perhaps in writing (mother of presymptomatic newborn, 2 SMN2).*
**EXPEDITING REFERRAL AND ACCESS TO SPECIALIST CENTRES**	Utilizing the role of the specialist multidisciplinary service	*We sat at home for several days with not much information and searched on the internet. Getting to see the specialist was a big relief as we could start doing something for our child (father of presymptomatic newborn, 2 SMN2).* *I really valued being referred to the neuromuscular team immediately. I could ask all my questions and because it's a rare disease, you really need the experts to guide you (mother of presymptomatic newborn, 3 SMN2).*
**OPTIMISING REPRODUCTIVE CHOICE**	Accessing prenatal/preconception screening methods	*It would have been better to know this [diagnosis] prior to conception or at least antenatally so we could adjust and plan for the future. In that respect, NBS does not go far enough (father of presymptomatic newborn, 2 SMN2).* *I think if we had known this sooner, this whole thing may have been avoided. Why can't you test sooner (father of symptomatic newborn, 2 SMN2)?*

HCP recommendations were categorised into four themes (Table 5). Of 15 HCPs, eight (53%) recommended *establishment of collaborative networks* between local, specialist and laboratory services. They perceived that defined pathways for referral streamlined the program and allowed professionals to share expertise, enabling a supported healthcare journey for the newborn and their family. Management of complex presentations were facilitated by discussion and goal setting within a multidisciplinary model of care for 4/15 (27%) HCPs. Navigating ways to ensure sustainability of the program, (prior to national dissemination) was important for six HCPs (40%). They emphasized the necessity of equipping a wider (non-specialist) workforce to deal with the demands of population screening in rare disease through education programs and access to expert opinions.

**Table 5:** Healthcare professionals’ recommendations on improvements to the pilot newborn screening program spinal muscular atrophy, with illustrative quotes.

Theme	Subtheme	Illustrative quotes
**ESTABLISHING COLLABORATIVE NETWORKS AND DEFINING REFERRAL PATHWAYS**	Accessing specialist resources	*Effective communication and collaboration between specialists and primary care clinicians is important to provide patient and family centred care at the first appointment. Telehealth for rural/remote patients can help in this (clinician).* *Rapid referral systems to specialist centres to undertake diagnostic testing and plan treatment facilitates good outcomes for those with a potential for early disease onset (clinician).*
	Establishing and supporting pathways between newborn screening laboratories and health services	*[*Newborn screening*] laboratories need clear cut guidelines on the clinical referral pathway whilst remaining flexible enough to facilitate a streamlined pathway for the newborn (clinician).* *Referral pathways need to be actively supported by a clinical genetics service so that results can be translated with a context (clinician).* *Coordination and communication between clinicians and laboratories were important, especially for unexpected results and to ensure rapid results (allied health professional).*
**DEVELOPING EXPERTISE OUTSIDE SPECIALIST CENTRES**	Facilitating ongoing professional development	*Having access to specialist expertise is great but as non-experts we also need maintain up to date information on this condition, especially as the evidence is changing so quickly. This way we can facilitate appropriate follow-up closer to home (clinician).*
	Access to educational tools	*We need to develop educational tools so that HCPs outside specialist centres feel supported in providing information and managing these children closer to home. This will help as we expand the NBS program out of the pilot phase (clinician).*
**OPTIMISING HEALTH PROVIDER COMMUNCATION**	Accessing written resources	*I think you need a written resource, perhaps in a range of languages so all parents are on the same page (clinician).* *It's difficult giving enough information without bombarding the parent. Access to an online resource may perhaps help them absorb the details and options at their own pace (allied health professional).*
**MANAGING COMPLEX PRESENTATIONS**	Utilizing the expertise of the multidisciplinary team to guide decision-making	*Having a multidisciplinary perspective of care was useful for me and for families. I think it provided both with an emotional and physical presence of support necessary following early diagnosis (allied health professional).* *Working as a team really helped me. Team discussions prior to seeing the family helped set goals of care (allied health professional).* *Teamwork/support from all disciplines helps overcome barriers where there is uncertainty as to prognosis (clinician).*

**Fig. 5:**
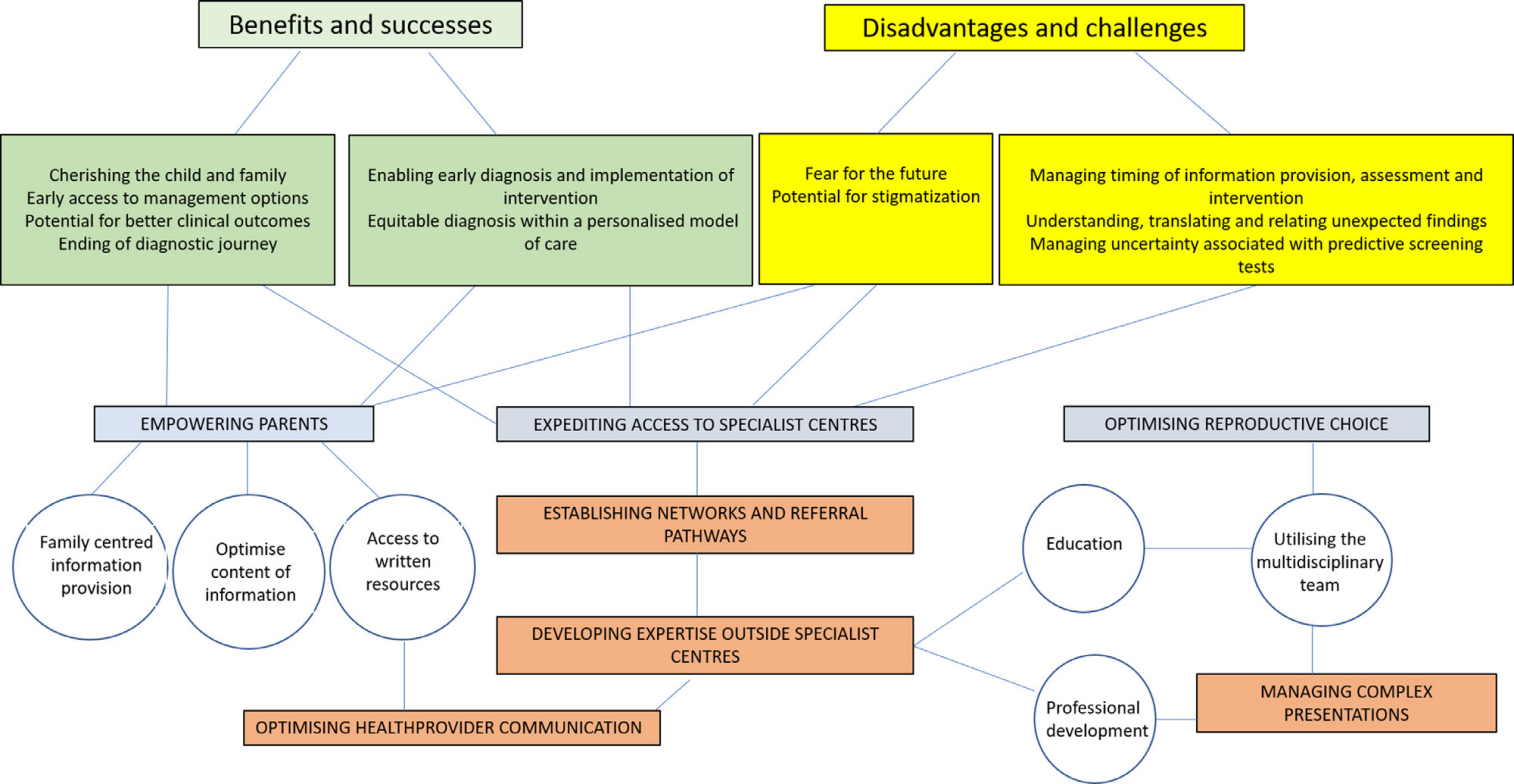
A thematic map displaying the interactions between perceptions and recommendations for parents’ and healthcare professionals involved in the newborn screening program for spinal muscular atrophy. Colour shadings for themes emerging from perceived benefits and successes of the program = green, perceived disadvantages and challenges of the program = yellow. Colour shadings for themes emerging from recommendations by parents = blue, themes emerging from recommendations by HCPs = orange, subthemes emerging from recommendations by parents’ or HCPs = circles.

A thematic representation of parents’ and HCPs perceptions of NBS for SMA and recommendations on how to address areas of need are shown in Figure 5.

## 4 Discussion

To our knowledge, this is the first study to evaluate perspectives of parents and HCPs within a NBS pilot program for SMA. Our study starts to address key implementational factors as advocated by modern screening principles [17,18] (consolidated on traditional criteria established by Wilson and Jungner) [19], determining that NBS for SMA is acceptable to stakeholders and facilitated by an approach that supports informed choice, autonomy and respect for the rights of the individual. Our findings emphasise that parents and HCPs acknowledge the disadvantages and challenges inherent to such programs, whilst concurrently recognising that the magnitude of benefits outweigh these barriers. The delineation and management of these harms and benefits guides policy decisions, facilitating development of infrastructure for population-wide screening, safeguarding sustainability of NBS for SMA and ensuring ongoing relevance and utility of this program for stakeholders [17].

We found that parents and HCPs unanimously supported the NBS for SMA. This finding is particularly relevant as satisfaction with (newborn) screening programs are essential for their successful continuance, with imposed programs being more susceptible to failure than those with strong consumer support [20]. Although NBS for SMA challenges traditional public policy on screening, due to a long latent phase in 20% of the prevalent population and paucity of long-term data on safety and effects of therapeutic intervention [21], our study determined that equitable access to NBS for SMA was advocated by those directly affected.

Perceived benefits of taking part in the program generally outweighed feelings of distress for parents. An early diagnosis was valued by parents as a gateway to accessing therapeutic intervention or surveillance with associated potential to improve health outcomes for their children. Interestingly, parents who chose not to treat their child with disease-modifying agents were similarly satisfied with participation in the program. These results have been replicated in NBS programs for neurodegenerative conditions such as Duchenne muscular dystrophy where there is no current recourse for disease-modification [22].

Consistent with previous literature and as expected, the inherent nature of receiving a true-positive result was universally distressing for parents [23,24]. Hypothetical concerns about a possible sustained negative impact on the family unit were not evident in our cohort within the first six months of life [11,25]. Improvement of QoL for our parents instead replicated studies of psychological sequalae associated with NBS for cystic fibrosis, where distress was temporary, however longer-term emotional impacts require further study [23]. Similar to findings from screening programs in cardiology and oncology, some parents acknowledged a sense of heightened fear for the future of their child after receiving a screen-positive result [26], thus psychological distress and reduction in QoL should be anticipated when new conditions are added to NBS programs. Adopting a biopsychosocial approach within healthcare practice is vital to ameliorate these ramifications and minimise disruption in familial well-being during a child's formative developmental period [27].

Poor recall and understanding of information and its perceived effect on parent distress is not a unique finding to our pilot and has been replicated across established NBS studies [28], [29], [30], [31], [32]. Finding the correct balance between eliciting undue concern in screen-negative families and providing sufficient information for the few screen-positive cases seen in rare diseases is a widely acknowledged challenge, requiring further interrogation [33]. Parents emphasised the requirement for a robust model of information provision, advocating a paced and individualised approach to fit their needs, alongside multimedia resources utilised as adjuncts to health provider communication.

As evidence for narrow therapeutic windows in SMA emerge [34], international guidelines concurrently advocate for intervention within two weeks of diagnostic confirmation in infants identified with two copies of *SMN2* and predicted to have a severe phenotype [35]. Regulation surrounding referral pathways between NBS, specialist and local health services are essential to provide streamlined access for the newborn and their family to best practice, whilst simultaneously supporting HCPs to facilitate this process.

Limitations of using predictive genetic testing as a screening tool are internationally recognised [36,37], and in clinical practice lead to difficulties prognosticating timing of disease-onset, relaying these uncertainties to parents and the potential creation of a ‘patient-in waiting’ population, as evidenced in our findings from HCPs. These challenges have been similarly noted in other NBS programs with late-onset disease phenotypes [38].

Although international expert consensus opinions advocate treatment for all newborns who screen positive for SMA with ≤4 *SMN2* copy numbers [6], a personalised model of care was advocated by HCPs and valued by parents, irrespective of treatment avenue pursued. These findings align with directives from NBS frameworks that advocate a balance between protecting a population's health and acting in the best interests of the individual [39,40]. HCPs particularly recognised the need to weigh long-term effects of the therapeutic burden for families, including the risks of overtreatment with invasive and lifelong therapeutics against benefits of early treatment. These perspectives may change as the therapeutic landscape in SMA rapidly shifts to accommodate an expanding repertoire of therapeutics, each with their own benefit/risk profile. The concurrent development of biomarkers of disease severity and treatment response will be vital to guide therapeutic management and create a patient focused model of care, as advocated by HCPs [41].

Public health programs should have the necessary infrastructure to facilitate long-term follow-up of screen-positive cases, whichever therapeutic avenue is pursued, in line with the aim of many NBS programs that advocate an integrated and long-term view of healthcare [39]. As we have recognised in our study, to facilitate this team-led model of healthcare, systematic training of HCPs is an integral part of healthcare provision, and has been effective in increasing understanding and engagement in NBS for conditions such as cystic fibrosis and metabolic disease [42]. While the Royal Australian and New Zealand College of Obstetricians and Gynaecologists recommend that carrier screening be offered to all women planning pregnancy or within the first trimester of pregnancy, barriers to equitable uptake include lack of awareness and the need to offer a publicly funded test [43].

Empowering parents of screen-positive newborns has been recognised as crucial to increasing compliance with treatment/monitoring and optimising health outcomes [42], and was advocated by both parents and HCPs in our study. Providing parents with instructive material supplementing and supporting communication aims to improve transmission of information and should potentially focus on the meaning and consequences of a positive result alongside intervention options [42]. This may be especially beneficial in diseases such as SMA where a rapidly evolving therapeutic landscape quickly leads to outdated communication.

Strengths of our study include perspectives gained from stakeholders, building a holistic view of the process. This includes whole population sampling for a rare disease and a high participation rate for parents and HCPs, encompassing parents of both genders and a spectrum of backgrounds. Therefore, our findings have the potential for applicability across a heterogeneous population.

Involving health care workers from different disciplines alongside parents helped to minimise potential bias. While caution regarding potential social desirability bias is needed, our mixed methodology incorporating qualitative data supported survey responses. There are, however, several limitations to consider when interpreting the study results. While no new themes were identified, indicating we achieved saturation, a larger sample size may have captured further depth of meaning: however, we believe the experiences derived from our sample are likely to reflect those of other parents with SMA newborns. The cultural and attitudinal factors arising from participants recruited from a single region, changing treatment options and ongoing implementation of SMA NBS into routine health practices may limit the generalizability of the results. However, many of the perspectives conveyed are likely to be relevant to those involved with SMA worldwide, serving to strengthen health systems and of broad interest.

This research has several implications for future policy and practice, providing evidence that NBS for SMA promotes equity in diagnosis, reaches agreement from the population for the test and indicates that successful implementation requires integration of education, testing and clinical services. As scientific evidence evolves and international best practice recommendations advance, findings from this study provide a unique foundation from which to ensure that real-world implementation of NBS programs for SMA minimise harm, whilst maximising benefits for screen-positive newborns and their families.

## Appendix Supplementary materials

Image, application 1
