# Supplementary material for: “We needed this”: perspectives of parents and healthcare professionals involved in a pilot newborn screening program for spinal muscular atrophy
Source: eClinicalMedicine. 2021 Feb 19;33:100742. doi: 10.1016/j.eclinm.2021.100742 (PMC8020144; doi:10.1016/j.eclinm.2021.100742)
Supplement: Supplementary file 1 [file mmc1.docx]

**Table S1. Study measures used to assess parents’ sociodemographic status and perspectives of benefits and satisfaction with the pilot newborn screening program for spinal muscular atrophy.**

| **Domain** | **Study measures** | **Response options** |
| --- | --- | --- |
| **Sociodemographic of parents** | Age, sex, highest educational level, employment status, religion, primary language, birth country, marital status |  |
| **Parents’ perceptions on benefits of NBS for SMA and satisfaction with participating in the program** | Parents’ satisfaction at each stage of the pathway and an overall satisfaction rating with the complete program (and justification of visual scale) | Visual scale of satisfaction where (0 = not at all satisfied and 100 = completely satisfied).  (Open ended question) |
|  | Parents’ beliefs on the benefit of participating in NBS for SMA at pre-screening | Extremely likely: > 90% chance of benefit, Very likely: 75-90% chance of benefit, Moderately likely to benefit: 50-74% chance, Somewhat likely: 25-49% chance, Very unlikely: < 10% chance of benefit and No chance of benefit. |
|  | Parents recommend the NBS program for SMA to relatives and would participate in future programs | Yes/No |
|  | Parents’ positive experiences at each stage of the program  ‘If you would recommend the program please explain why’ | Open ended question |
|  | Parents feelings after receiving screen positive and diagnostic test results | 1. ‘reassured/confident’, 2. ‘angry’ 3. ‘sad’, 4. ‘confused’ 5. ‘worried’ |
|  | Change in quality of life between receiving a scree positive/diagnostic result, six and twelve months from diagnosis | (CarerQoL-7D), constitutes an eight item questionnaire that assesses the caregiver’s fulfilment with care tasks, relational problems the newborn, problems with the parents mental health, problems combining care tasks with daily activities, financial problems secondary to care tasks and physical problems (response options: ‘no’, ‘some’ and a lot of’, ) The eighth item is a self-rated happiness today with a visual analogue scale from 0 to 10 (where 0 is completely unhappy and 10 is completely happy). |
|  | Parents’ negative experiences at each stage of the program  ‘If you were not satisfied please explain why.’ | Open ended question |

**Table S2. Study measures used to assess parents’ perspectives on information provision, health professional communication, access to support and recommendations for service improvement.**

| **Parents’ perceptions on the quality, content and clarity of information provision** | Timing, modality of information provision and role of information provider at pre-screening  Parents’ perceived understanding of what the screen was trying to detect and why it was important, overall understanding of NBS content and process for newborn.  Parents’ perception of screen and diagnostic result information being clear or sufficient | Five-point Likert: ‘I understood this very well’ to ‘I didn’t understand at all.’  Stratified into: I understood this very well/I understood this well (1) and I understood this somewhat/ a little / I did not understand at all (2) |
| --- | --- | --- |
|  | Parent recall of neuromuscular disorder being discussed at pre-screening | Yes/No |
|  | Perceptions on how information provision could be improved at each stage of the pathway  Reasons that parents perceived that information provided was either unclear or insufficient | Open ended questions |
| **Parents’ perceptions on quality of healthcare provider communication** | Parents’ perception on sensitivity, clarity of health provider communication  Parents’ perceptions on opportunity to ask questions and the appropriate answering of queries | Five-point Likert: ‘Extremely true’ to ‘Not at all true.’  Stratified into: (1) Extremely and very true (2) and somewhat/a little/ not true at all |
| **Parents’ perceptions on timely and appropriate access to healthcare resources** | Parents recall of being provided with written resources, a designated point of contact and access to the multidisciplinary team (at diagnostic stages) | Five-point Likert: ‘Extremely true’ to ‘Not at all true.’  Stratified into: (1) Extremely and very true (2) and somewhat/a little/ not true at all |
| **Recommendations on how to address unmet areas of needs (including information and service needs)** | At each stage of the program, we asked parents ‘How could your experience be improved’ | Open ended question |

**Table S3. Study measures used to assess healthcare professionals’ perspectives of the pilot newborn screening program for spinal muscular atrophy.**

| **Domain** | **Study measures and items** | **Response options** |
| --- | --- | --- |
| **HCP background and role within the newborn screening program for SMA** | Professional title, time spent in this field and previous experience in managing children with SMA and the details thereof | Open ended question |
| **HCP perceptions on successes and challenges of healthcare provision within NBS for SMA** | Negative and positive experiences as associated with their role in the newborn screening program for SMA  Barriers/challenges to performing their role and any helpful measures used to overcome these barriers.  Perceived benefits and risks of NBS for SMA  Differences in disclosing results/information arising from NBS for SMA compared with other medical results | Open ended questions |
| **Recommendations on how to address unmet areas of needs (including information and service needs)** | HCP recommendations on how to improve any aspect of the NBS program for SMA | Open ended question |
